# Complete Genome and Plasmid Sequences of Salmonella enterica subsp. *enterica* Serovar Enteritidis PT1, Obtained from the *Salmonella* Reference Laboratory at Public Health England, Colindale, United Kingdom

**DOI:** 10.1128/MRA.01064-19

**Published:** 2020-01-09

**Authors:** Muhammad Zohaib Anwar, Athanasios Zervas, Lars Hestbjerg Hansen, Gary Barker, Alexandre Magno Anesio, Carsten Suhr Jacobsen

**Affiliations:** a Department of Environmental Science, Aarhus University RISØ Campus, Roskilde, Denmark; b Department of Plant and Environmental Sciences, University of Copenhagen, Copenhagen, Denmark; c School of Biological Sciences, University of Bristol, Bristol, United Kingdom; University of Maryland School of Medicine

## Abstract

Hybrid assembly of Illumina and Oxford Nanopore sequencing was used here to produce the complete circular genome and plasmid sequences of Salmonella enterica subsp. enterica serovar Enteritidis PT1 (phage type 1). The organism was obtained from the *Salmonella* Reference Laboratory at Public Health England, Colindale, UK.

## ANNOUNCEMENT

*Salmonella* members are classified into >2,500 serovars based on the Kauffmann-White scheme (1). Salmonella enterica subsp. enterica serovar Enteritidis is associated with salmonellosis in humans and domestic animals (1). Salmonella enterica subsp. *enterica* serovar Enteritidis has been frequently detected as the causative agent in foodborne outbreaks (FBOs) reported in Europe and North America (2–4). The complete genome sequence of this strain will not only serve as a reference for identifying similar outbreaks with more precision in the future but also aid in the identification of similar organisms in environmental samples. Salmonella enterica subsp. *enterica* serovar Enteritidis PT1 was grown at 37°C overnight in LB growth medium. Three samples were taken from one liquid culture and were used for individual DNA extractions. DNA was extracted from 200 μl of each sample using the AllPrep PowerFecal DNA/RNA kit (Qiagen, Hilden, Germany). Short-read sequencing libraries were prepared using the Illumina TrueSeq DNA PCR-free high-throughput library prep kit. Libraries were pooled equimolarly and sequenced (single-end 75 bp) on the NextSeq platform using the NextSeq 500/550 high-throughput kit v2.5 (Illumina, San Diego, CA, USA). A total of 94,938,911 single-end 75-bp reads were subjected to quality trimming and sequence contamination removal (–average_qual < Q20) using fastp (5). High-molecular-weight DNA for Nanopore sequencing was also extracted from 1-ml cultures using the MasterPure complete DNA and RNA purification kit (catalog number MC85200; Epicentre, Lucigen), according to the manufacturer’s instructions. Libraries for Oxford Nanopore sequencing were prepared using the rapid sequencing kit (SQK-RAD004) and loaded on a MinION flow cell (R.9.5.1). Sequencing was stopped manually after 22 hours when 6.44 Gb were obtained, resulting in 359,803 long reads with an *N*_50_ value of 30.23 kb. Adapters were trimmed from long reads using Porechop (https://github.com/rrwick/Porechop). Trimmed Illumina and Nanopore reads were then used for a hybrid assembly using Unicycler v0.4.4 (6), which uses SPAdes v3.13.1 (7) for short-read assembly and miniasm and minimap (8) for long-read assembly and mapping the bridges with short reads, respectively, and Racon v1.4.0 (9) and Pilon v1.23 (10) for polishing the assembly. The complete genome sequence (100% coverage) consists of one circular chromosome (4,685,587 bp) with an overall G+C content of 52.17% and one circular plasmid sequence (59,372 bp) with a G+C content of 51.95%. The genome and plasmid were annotated using rapid prokaryotic genome annotation (Prokka) v1.13.7 (11) with genome and plasmid parameters, respectively. A total of 4,463 predicted genes were identified on the genome, including 22 rRNA, 84 tRNA, 1 transfer-messenger RNA (tmRNA), and 4,354 protein-coding genes, while 75 coding DNA sequences (CDSs) were identified on the plasmid. The genome was then compared with 6 other Salmonella enterica genomes and the Sphingobium herbicidovorans MH (12) genome based on marker gene lineage analysis using CheckM v1.0.7 (13), which indicated 100% completeness of the genome and 0.00 strain heterogeneity with Salmonella enterica subsp. *enterica* serovar Enteritidis. All bioinformatics software programs were used with default parameters, unless otherwise stated.

### Data availability.

Illumina short reads were deposited at the SRA and are available under BioSample accession numbers SAMN12634776, SAMN12634778, and SAMN12634782 under BioProject accession number PRJNA562128. Oxford Nanopore long reads were also deposited at the SRA and are available under BioProject accession number PRJNA562126. The complete genome and plasmid sequences are available at GenBank under accession numbers CP043433 and CP043434, respectively, and under BioProject accession number PRJNA562154.
